# Medico-Chirurgical Transactions, Published by the Royal Medical and Chirurgical Society of London. Second Series, Volume the Eighth

**Published:** 1844-04-01

**Authors:** 


### Medico-Chirurgical Transactions, published by the Royal Medical and Chirurgical Society of London. Second Series, Volume the Eighth. 8vo. pp. 425. London, 1843.

[Concluding Notice.]

In our last Number, we gave an account of many of the Papers contained in the volume before us. We shall now bring that account to a conclusion.

### I. A Few Observations on Encysted Hydrocele. By *Robert Liston*, Esq. F.R.S.

Mr. Liston's object is to point out the possible connection of encysted hydrocele with some of the seminiferous tubes of the testicle. After observing that the collections forming encysted hydrocele are described as occurring,—

"I. On the testicle, betwixt the albuginea and tunica vaginalis—at first as small transparent cysts, but gradually increasing in size.

"II. As presenting by the side of the epididymis, betwixt that body and the reflection of the tunica vaginalis from the testis.

"III. As appearing in the course of the spermatic chord above the testicle. In this latter situation, no doubt, collections of various kinds are to be met with in the loose filamentous tissue of the chord; in the unobliterated portions of the spermatic process covering that body; or, possibly, in more immediate connexion with the vas deferens itself." 217.

Mr. Liston goes on to say, that the fluid of encysted hydroceles of the chord, varies, as might be expected in quality, from an albuminous to a limpid fluid—that, when the latter is the case, it is well known that the cyst does not become as readily obliterated by injections, &c. as do serous cysts—and that, the object of the present communication is to explain why this should be.

A gentleman had a cyst connected with the testicle punctured, and the fluid drawn off. On the third occasion scarcely a trace of albumen could be detected.

"On the second day, a minute quantity was put in the field of the compound microscope, and my surprise was great indeed when it appeared quite full of spermatozoa—there were besides to be detected some of the primitive cells, in which the spermatozoa are developed, and a certain number of mucous globules." 219.

The fluid not being examined immediately, it was not ascertained whether the animalcules had their usual liveliness of motion. Mr. Liston has been making inquiries upon the subject, and he finds that, in some cases, "cysts are formed behind the testis; others in the fore part, projecting into and covered by the reflected tunica vaginalis, and no doubt by the tunica albuginea also; other, and in some instances, numerous cysts are seen above the testicle—multilocular hydrocele of the chord." There is a specimen in the collection at St. Bartholomew's Hospital, without any history, but described, in the catalogue, thus:—

"'Testicle with part of the spermatic chord; along the epididymis there is a series of membranous cells communicating together, and having for their outer boundary the tunica vaginalis, and its reflexion between the testicle and epididymis. *These cells contained a transparent and colourless fluid.* A bristle is passed beneath the vas deferens near its connexion with the epididymis.'

"Here the sac is closely connected with the epididymis, *if not an actual dilatation of its lesser head.**

---

* "There is also a preparation in the collection of Mr. Bransby Cooper, in which a cyst is seen connected with the upper part of the epididymis, pushing the tunica albuginea of the testis before it, and projecting into the cavity of the tunica vaginalis. Mr. Cooper punctured this cyst, and drew off three or four ounces of limpid fluid, which contained scarcely a trace of albumen. The patient died of pneumonia a few months after the operation, and the preparation being obtained, showed the sac somewhat contracted, though gradually refilling."

"This subject deserves further investigation, to discover, first, if the limpid fluid drawn from cysts of the scrotum and inguinal region, uniformly or often contains spermatozoa.

"Secondly, what connexion subsists betwixt the seminiferous tubes and their cysts.

"Thirdly, whether or not dilatation of parts of the epididymis or vas deferens, obstruction or otherwise, may not, in some instances, give rise to these collections.

"If so, this being a pouch lined by mucous membrane, we should have an easy solution of the difficulty regarding a radical cure, not following injection as in the serous cyst. The microscopic examination of the lining membrane of a recent cyst would easily settle the nature of the secretory surface." 221.

In a case which has subsequently occurred at the Hospital, the fluid from a small cyst above the testicle of a man, aged 53, was nearly transparent and colourless, and contained spermatozoa which moved actively for some time.

In November last, Mr. Dalrymple again drew the attention of the members of the Society to this subject, and offered a new explanation of the occurrence of spermatozoa in the fluid of hydrocele. "He quoted the authority of Scarpa to show that when the tunica vaginalis becomes distended with fluid the different vessels of the spermatic chord are separated from each other to a greater or less distance, owing to the gradual expansion of the membrane, and the epididymis and vas deferens are, consequently, removed out of their natural places in reference to the body of the testicle. He likewise presented to the society a preparation, with a drawing of a hydrocele, in which that condition of the tubes of the testis was exhibited."* In fine, his opinion was, that the epididymis and vas deferens *might* be punctured in the operation of tapping, and that even a single tube wounded might afford the few spermatozoa found in common hydrocele.

In the present Volume of the Transactions, there is another paper on the "Presence of Spermatozoa in the Fluid of Hydrocele," by Mr. Lloyd. He first observed the animalcules in operating on what "there was no reason to doubt" was "hydrocele of the tunica vaginalis testis." The spermatozoa were numerous. "The fluid of the hydrocele, in the precise condition in which it was abstracted, contained, in addition to the spermatozoa, a few blood globules, small roundish granular bodies, some apparently empty, nearly colourless cysts, and many masses of opaque matter, which seemed to be made up of portions of epithelium." Mr. Lloyd has subsequently examined the fluid in about thirty cases, and found living spermatozoa in two. In the second case, one of common hydrocele, with the fluid highly albuminous, there were likewise a few blood globules, transparent cysts, and small granular bodies; also portions of epithelium, or what very much resembled it.

In the third case, "the situation of the fluid appeared to be very much that of common hydrocele of the tunica vaginalis, and after the operation had been performed there was nothing peculiar to be observed in the testicle or its appendages. There were about four ounces of fluid abstracted,

---

* Lancet, Dec. 16, 1843.

which was of paler colour than is usual in hydrocele, and displayed very much the appearance described by Mr. Liston as exhibited by the fluid of the encysted hydrocele, in which he had discovered spermatozoa. But in one respect it differed from that, as it contained a considerable quantity of albumen. There was also found in it much saline matter; but the precise nature and proportions of which were not ascertained. The spermatozoa met with in the fluid were very numerous" and living. Here too, the fluid contained a few blood globules, transparent cysts, spermatic granules, and scales of epithelium. This patient has been tapped a second time with the same results.

In the "Transactions," Mr. Lloyd professes himself unable to explain the phenomenon. But in the discussion on Mr. Dalrymple's Paper, to which we have alluded, he takes courage, and is of opinion that either a previous abscess of the testicle may have been the source of the spermatozoa, or that they may be formed by a "vicarious action." This certainly does not explain much, and must be looked on as rather hypothetical at present; and, in fact, we must conclude that the subject needs more investigation. For ourselves, we are satisfied that parts of the seminiferous apparatus, not excluding the testicle itself, *are* occasionally wounded in the operation for hydrocele, and *may* account for the spermatozoa in some instances. Whether it will do so in all is problematical.

### II. STATISTICS OF BETHLEM HOSPITAL, WITH REMARKS ON INSANITY. By *John Webster*, M.D.

Dr. Webster has conferred a material favour on the profession by the pains he has been at in arranging the data of the present communication, and by the careful manner in which it has been compiled. It comprises a statistical report of the principal occurrences met with at Bethlem Hospital, during the last and present century. We are presented with the number of admissions and of deaths, and the proportion of patients reported cured, during different periods, of equal duration. These classified statements are accompanied by a few general remarks on insanity; and, lastly, an account is given of the chief pathological changes of structure observed on the dissection of seventy-two insane patients, which have been recently performed in that hospital.

It appears that, since the year 1683, 22,897 insane patients, exclusive of incurable and criminal lunatics, have been admitted into Bethlem Hospital, but the present paper refers only to the occurrences of the last hundred years.

"In pursuing this investigation, the number of patients received into Bethlem Hospital, with the total amount of cures, and the actual deaths reported, as also the per centage calculated on each, will be enumerated under separate heads; embracing, however, different periods of twenty years in each division; the first commencing the 1st of January, 1743, and the last terminating the 31st of December, 1842. It is, nevertheless, right to mention, that, owing to defects in some of the official registers of the institution, the exact number of patients discharged cured from Old Bedlam, and the amount of deaths which took place in one or two of the years prior to 1748, could not be accurately ascertained, and are therefore given from a comparison with the results of subsequent years." 376.

The number of admissions reported are, however, correct, as well as the other particulars in the Tables. The first of these is a—

*Table exhibiting the total number of lunatic patients admitted into Bethlem Hospital, discharged cured, or died, during five different periods of twenty years each, ending the* 31*st December respectively.*

Without inserting the Table itself, we will advert to the main features of it, and to the deductions of our author. In each period of 20 years, with the exception of those ending in 1822, and in 1842, the number admitted was between 3 and 4,000. But in the former of those last-named periods, the admissions decreased to 2,149; and in the latter, they rose to 4,404.

The material diminution in the number of patients admitted, during the first half of the present century, must be looked on as an accidental circumstance, for the number of patients received during the same period into St. Luke's Hospital was greater than during the succeeding one.

But a gratifying fact is, the uniformly increasing proportion of patients discharged cured, and the diminution in the number of deaths, since the middle of the last century.

"For instance, the ratio of recoveries during the first twenty years embraced in the preceding statement, was only 32¼ per cent. on the admissions, whilst it rose to 51½ in every hundred patients admitted during a similar number of years ending the 31st December, 1842; at the same time that the amount of deaths actually decreased from 21⅔ per cent. to 5$\frac{1}{14}$ per cent., or less than one-fourth the previous average, after an interval of nearly a century. The difference will, however, appear even more marked, when the results met with during three years in the middle of the last century, are compared with similar results reported to have occurred in the three years just terminated. In illustration of this point, it is only necessary to refer to the official tables already quoted, which show that the number of insane patients received into Bethlem Hospital, discharged cured, or died, during the specified period, were as follows:—

| | ADMITTED. | CURED. | DIED. |
|---|---|---|---|
| In 1750–51–52, | 462. | 145 or 31½ per cent. | 118 or 25½ per cent. |
| In 1840–41–42, | 897. | 492 or near 55 per cent. | 51 or 5⅝ per cent." |

In 1753 the deaths at Old Bethlem Hospital exceeded the recoveries, but then the small-pox prevailed. In 1770, another year of small-pox, the deaths and recoveries were equal.

Women are more prone to insanity in this country than men. During the twenty years ending December 31, 1842, the following were the numbers of admissions, cures, and deaths in each sex, in Bethlem Hospital.

| ADMITTED. | | CURED. | | DIED. | |
|---|---|---|---|---|---|
| M. | F. | M. | F. | M. | F. |
| 1,782 | 2,622 | 823 | 1,446 | 112 | 112 |
| or 47 per cent. more females than males. | | or 46⅕. per cent. | or 55⅐ per cent. | or 6¼ per cent. | or 4¼ per cent. |

Dr. Webster observes that "the excess of insane women admitted at Bethlem Hospital is shown to have been 47 per cent., and, as the same facilities regarding the admission of patients into that institution prevail, without any reference to sex, provided the cases are recent, the above results must be considered conclusive. A similar opinion is likewise fully borne out by the number of insane patients of each sex admitted into St. Luke's Hospital, during the same period of twenty years, to which reference has just been made. By returns obtained through the kindness of a friend, it appears that 1,734 lunatic male patients were received into the wards of that charity, from the 31st December 1822, to the 31st December 1842; whilst the number of insane females admitted during the same period, amounted to 2,310, or 33⅓ per cent. more of the latter than the former sex. According to these data, derived from the only two public institutions for the insane in London, and therefore the best guides to follow in making any calculations upon the subject, it may be stated that mental affections are much more frequently met with among women than men, at least in this part of the empire; where, speaking generally, the difference amounts to about 35 or 40 per cent. at an ordinary estimate."

Insanity, however, is much more curable in women than in men, and much less fatal to the former. In the table just given, the ratio of recoveries from attacks of insanity was nine per cent. in female more than in male patients; whilst in the latter sex, the number of deaths exceeded by two per cent., the rate of mortality met with in the former. The same fact is noticeable among the insane patients classed as incurable.

"Thus, on that list, 70 male, and 80 female lunatics were admitted into Bethlem Hospital, during the twenty years ending the 31st of last December, and although all were considered confirmed maniacs at the time of their reception, 18 women were actually discharged cured, whilst only six men in the same class of patients left the house convalescent. The results being, that 22½ per cent. of the incurable female patients recovered, and only 8$\frac{4}{7}$ per cent. of the male lunatics similarly affected. It is also interesting to mention, that the average deaths were even less numerous among the incurable women than men, of whom 31 patients died, being at the rate of 44$\frac{2}{7}$ per cent.: whilst among the incurable females, 34 died, or 42½ per cent. upon the admissions. From which data it consequently appears, that among 100 incurable female lunatics similarly affected, there were two deaths less than among the same number of incurable male patients." 383.

The physician may, with confidence, as Dr. Webster observes, offer, cæteris paribus, a more favorable prognosis in the case of a female than of a male lunatic.

A suicidal tendency is too much a feature of insanity, to permit those who have the charge of these patients to lose sight of it. The greatest care should be observed. But suicide, amongst the insane, is much less frequent than it was, notwithstanding the increase of liberty enjoyed by them.

"On this point, some interesting information may be obtained from the registers of the hospital alluded to; in which it is stated that suicides were more common in that charity during the last, than they have been in the present century. By these records it appears that 18 cases of self-destruction occurred in old Bedlam, from the 1st of January 1750, to the 1st of January 1770, six being male patients, and twelve female; and as the total number of lunatics admitted into that hospital, during the above period, amounted to 3,629 patients, there consequently occurred one suicide in about every 202 admissions."

But, "during the twenty years ending the 31st of December 1842, notwithstanding the total admissions, including 150 incurable, and 122 criminal lunatics, amounted to 4,676, only five suicides occurred in this royal hospital; which gives one case in every 925 insane patients admitted, or less than one-fourth the average number met with among the lunatics confined in old Bedlam, during the middle of the eighteenth century." 386.

It is worth noticing that all the five instances were those of women. This, with the fact already stated, shews a greater tendency to self-murder on the part of females than of males.

Another circumstance which tells strongly in favour of the humane management of the insane, is the comparative infrequency of escapes at present. It appears that, during the 20 years ending the 1st of January 1770, to use the expressive words met with in these official documents, 44 male and 11 female lunatics actually 'ran away' from the hospital, during that period; being one escape in every 66 patients admitted. But the escapes during the twenty years ending the 31st December 1842, were 11 men and 5 women, being only one evasion in every 292 admissions, or less than one-fourth the previous amount. Considering however, that as 14 of the patients who escaped were afterwards brought back to the hospital, in reality, only two lunatics permanently succeeded in their attempt. The escapes (the reverse of what was observed of self-destruction) have always been more numerous amongst the males. But it seems to us that this is explicable on obvious grounds. Men are more adapted, from habits of life, their dress, &c., for scaling walls, evading pursuit, &c., than women are.

The longevity, or otherwise, of the insane is an interesting question. On this head, the statistics of Bethlem do not afford much evidence of consequence. But it would appear that insane females are more likely to attain old age than are the males:—

"For example, among the incurable men now in that establishment, seven were inmates prior to 1830, and have been so ever since; whilst on the female side, seventeen incurable patients come within a similar category. Of the latter sex, one patient has continued insane upwards of fifty years; forty-nine of which she has constantly passed in Bethlem Hospital; another female lunatic has been insane for forty-five years, and an inmate of the charity during thirty-six; and a third individual belonging to the same class has been in the asylum during thirty-eight years, having lost her reason a year before. On the other hand, although the results met with among the incurable male lunatics are somewhat different, the facts nevertheless show, that the opinion previously expressed respecting the patients' prospect of longevity, notwithstanding the existence of insanity, is supported by ample experience. Thus, of the seven male lunatics reported insane previous to 1830, one has been an inmate of Bethlem Hospital during forty-three years, another thirty-eight, and a third for the same period; but respecting the length of time they have been actually insane, the registers are silent." 389.

Dr. Webster communicates a valuable synopsis of seventy-two dissections of lunatic patients, recently made at Bethlem Hospital, by Mr. Lawrence. The account of the autopsies is derived from the written reports of that gentleman in the Register. We must content ourselves with adverting to the conclusions drawn by Dr. Webster, recommending our readers who are interested in the matter to consult the synopsis in the original.

The dissections referred to are not selected, but given in the order in which they occurred, two only having been omitted. One of these was of a female patient who died of strangulated femoral hernia—the other of one who died of diseased lungs. In neither was the head examined.

" Some pathological changes of structure, more or less evident, were found in the brain or membranes of the whole seventy-two dissections reported, of which it may be stated, as a summary, that 55 cases likewise exhibited diseased alterations of structure of some kind or other in the organs of the chest, whilst only 14 patients showed any morbid appearances in the abdominal viscera." 412.

It must however be stated, that the abdomen was not opened in every instance, which, of course, throws some doubt upon the subject. But to proceed.

" In 59 cases, there was infiltration of the pia mater. In 59, turgidity of the blood-vessels of the brain and membranes. In 41, effusion of water in the ventricles. In 27, water was met with at the basis of the brain. In 19, bloody points on the cut surfaces of the medullary substance. In 16, thickening and opacity of the arachnoid coat. In 14, the colour of the medullary or cortical substance of the brain was altered from its natural hue to brown, pink, grey, violet, ochre, or white. And in 13 cases, there was an effusion of blood in the brain. Besides these diseased appearances, various other alterations of structure were met with in particular patients; such as effusion of pus on the brain; changed consistence of its texture; greater dryness than usual of the membranes ; flattening, a shrunk, or a swollen state of the organ itself; with other changes different from a normal condition; for an account of which I would refer to the synopsis, to avoid superfluous repetition." 413.

We would observe that the ages of the patients, at the time of their death, is not mentioned. This seems to us a material omission, in estimating the value of the pathological change. Many of those set down in the synopsis are such as are commonly met with in persons advanced in life, independently of derangement of the intellectual functions—subarachnoid effusion and thickening—turgidity and congestion of the cerebral veins.

" Although diseased alterations of structure were not so frequently met with in the organs of the chest, as in the brain and its membranes, nevertheless, 55 insane persons in the above list of dissections exhibited changes of a morbid description in the thorax. Indeed, the apparent cause of death, in many of the patients, could be clearly traced to disease in the organs of respiration. Of the 55 instances of pectoral disease met with, on examining the bodies after death, 43 cases showed either recent or old adhesions in the chest, and 31 had the lungs consolidated. In 24, suppuration had commenced. In 15, the pleura or lungs bore marks of recent or previous inflammation. In 12 cases, there was effusion of lymph into the pleura, &c. In 9, considerable effusion into the bronchi and air-passages. In 9, the lining membrane of the trachea and bronchi was deep red. In 8, tubercles were met with. In 6, the lungs had assumed a dark, or blackish tint. And in 7, the lungs did not collapse when the chest was opened." 414.

The alterations of the abdominal viscera were not numerous. "The liver was found to be affected in five cases; dropsical effusion had taken place in three patients ; and in three other cases, there appeared decided marks of recent and violent inflammation of the contents of the abdomen; in two of which examples, the intestines had actually given way, so as to allow fæcal matter to escape into the peritoneal cavity ; and a similar result would have likely supervened in the other case of intestinal inflammation, had the patient lived for a longer time."

Dr. Webster indulges in few remarks. His opinions are so briefly stated that we transcribe them.

" Considering the present communication to have already much exceeded the limits originally proposed, I will not attempt to discuss cursorily the important question which now occupies many pathologists, regarding the rationale of the diseased appearances usually met with in the brains of lunatics on dissection after death, namely, whether the morbid alterations of structure, then observed, be the cause, or only the consequence of the patient's previous mental malady. In short, whether the opinions promulgated by the section of pathological physicians, denominated 'the Anatomists,' or the views entertained by the other party, 'the Vitalists,' be the true doctrine. The former considering that the diseased alterations of structure observed in the brain produce the attacks of insanity: whilst the latter confidently assert the contrary. I therefore shall leave the decision of such important questions to others more competent than myself to give an opinion; although I must confess, the numerous illustrations now detailed, the facts recorded in medical works, as well as the reasoning of authors upon insanity, greatly preponderate in favour of the Anatomists; whose conclusions, in my judgment at least, appear to be the most rational, and quite consistent with the present state of our pathological knowledge respecting mental diseases." 416.

It certainly appears to us most rational to conclude, that, as the brain is the undoubted organ of the intellectual functions, they cannot be permanently deranged without *some* alteration of its structure. But morbid anatomy has not yet succeeded in revealing to us, in the early stages of insanity, what those alterations are. No doubt, they are sometimes appreciable enough—but, at other times, they are far from satisfactory. This can occasion no surprize when we reflect on the consequences of concussion of the brain—the insensibility, the interference with the intellectual functions, nay, the death itself of the patient, without evident cerebral lesion. The phenomena of syncope and epilepsy shew the close dependence of the brain upon the circulation, and leave us to imagine how slight disturbance of the latter in its substance may affect its functions. All these considerations tend, in our opinion, to encourage the belief, that, when in cases of insanity, we find no changes of structure in the brain, there may still be some of too delicate a nature to be recognised by us. And the serous accumulations, the turgescence of the venous system, and the other lesions within the cranium, so generally present in old cases of insanity, rather go to prove that the changes have advanced to more tangible degrees, than that these are merely the results of morbid action, not essentially affecting structure. Such views are borne out by the valuable paper before us.

### III. Second Series of Observations on the Pathology of the Ear. Based on One Hundred and Twenty Dissections of that Organ. By *Joseph Toynbee*, F.R.S.

A former Paper of Mr. Toynbee's was intended and calculated to prove that the lining membrane of the tympanitic cavity is frequently in a diseased condition. Subsequent researches have convinced him "that the most prevalent cause of deafness is chronic inflammation of the mucous membrane which lines the tympanic cavity; and that by far the greater majority of cases commonly called nervous deafness ought more properly to be attributed to this cause. This opinion derives support from an observation made to me by Mr. Swan, that in the whole course of his multiplied aural dissections he has not encountered one single instance of disease in the internal ear; an observation which embodies the result of repeated examinations to which I have myself subjected that part of the organ."

His object, in the present paper, is to elucidate the different stages of this disease of the mucous membrane, and to point out the various morbid conditions to which it gives rise. He gives the dissections more or less in detail, that the facts themselves may be judged of. He remarks that it is singular that the greater number of the patients, when living, were not supposed to be deaf.

This cuts two ways—it may shew that deafness, to some extent, is often overlooked—or it may be that these lesions of the tympanum do not necessarily occasion much defect of hearing. Be that as it may, the frequency and the nature of the lesion ought to be rightly understood.

The first point to which Mr. Toynbee alludes is—

*Inflammation of the Mucous Membrane lining the Cavity of the Tympanum.*

After giving a concise but ample account of the natural state and distribution of the mucous membrane of the tympanum, Mr. Toynbee goes on to observe that the changes produced in it by inflammation may be divided into three stages.

In the *first*, "the membrane retains its natural delicacy of structure, though its blood-vessels are considerably enlarged and contorted, and blood is effused into its substance, or more frequently at its attached surface. Blood has also been found between the membrane and the membrana propria of the fenestra rotunda, and in very acute cases lymph is effused over its free surface." 303.

The *second stage* is characterised by a variety of important phenomena, of which the principal are:—

"1st. A very considerable thickening of the substance of the membrane, which is often pulpy and flocculent. In this state the tympanic plexus of nerves becomes concealed; the base and crura of the stapes are frequently entirely imbedded in it; while the fenestra rotunda appears only like a superficial depression in the swollen membrane. Occasionally there is also a collection of mucus.

"2nd. Concretions of various kinds are visible on the surface of the thickened membrane. In some cases these have the consistence of cheese, and are analogous to tuberculous matter; in others, they are fibro-calcareous, and exceedingly hard.

"3rd. But by far the most frequent and peculiar characteristic of this second stage of the disease, is the formation of membranous bands between various parts of the tympanic cavity. These bands are at times so numerous as to occupy nearly the entire cavity. They are found connecting the inner surface of the membrana tympani to the internal wall of the tympanum; to the stapes, and to the incus. They have also been detected between the malleus and the promontory; as well as between the incus, the walls of the tympanum and the sheath of the tensor tympani muscle; and they so connect various parts of the circumference of the fenestra rotunda, as to form a network over the membrana propria. But the place where these adhesions are most frequently visible, is between the crura of the stapes and the adjoining walls of the tympanic cavity; this, for example, was the case in twenty-four instances out of a hundred and twenty dissections—being a fifth of the number. In one dissection, the bands of adhesion were five in number: and in other instances they were so strong, that, in removing the stapes, the mucous membrane was torn from the surface of the promontory. Sometimes so broad and expanded have been these adhesive bands, as to have assumed the appearance of a membranous veil. They have also been known to contain blood and scrofulous matter. In some examples the surface of the promontory is rough, and in two instances the membrane attached to the base of the stapes was ossified, and the anchylosis of the latter to the fenestra ovalis was complete." 305.

On these facts Mr. Toynbee makes several judicious observations. He naturally thinks it impossible that many of the preceding conditions can exist without more or less functional derangement of the organ of hearing. The thickening of the membrane and deposition of mucus must interfere with the course of the vibrations towards the fenestra rotunda, and with the action of the stapes.

"The bands of adhesion connecting the stapes with the walls of the tympanum, cannot do otherwise than impede the natural movements of the former, which has very frequently been found so firmly attached to the fenestra ovalis, as to require considerable pressure with the scalpel to disengage it. Morgagni states, that he found the cavity of the tympanum intersected by numerous membranes, which impeded the movements of the ossicula; and it appears highly probable, that these bands of adhesion produce irregular movements in the ossicula. I am inclined to ascribe deafness, and many of the distressing symptoms that often accompany it, as noises like the rushing of waters, &c. &c., to the continued pressure exerted on the contents of the labyrinth by the stapes being drawn inwards, as a consequence of the formation and subsequent contraction of the adhesions. In this opinion I have been strengthened by the examination of living persons, having frequently observed, that where the membrana tympani has been removed by disease, or where the contents of the vestibule have not received any impression through the stapes (as in the instance of the latter bone being anchylosed), the patients have heard better than those where satisfactory evidence existed, that the disease consisted in the thickened and adherent state of the membrane under consideration." 306.

The firm bands of adhesion connecting the membrana tympani to various parts of the tympanic cavity, with the implication of the muscles, must exert an influence more or less prejudicial, on the state of the membrana tympani, quoad its state of tension, which cannot fail to be injurious.

"*In the third stage* of inflammation of the tympanic mucous membrane, it becomes ulcerated, the membrana tympani is destroyed, and the tensor tympani muscle atrophied. The ossicula auditus are diseased, and ultimately discharged from the ear, and the disease not unfrequently communicates itself to the tympanic walls, affecting also the brain and other important organs." 307.

Mr. Toynbee appends an account of the dissections on which the preceding generalizations were founded. For these we must refer to the original, contenting ourselves with observing that, of 120 dissections there were:

20 Ears in the first stage of inflammation of the tympanic cavity.
65 Ditto in the second stage.

6 Ditto in the third stage.
29 Ditto in a healthy state.

We trust that Mr. Toynbee will continue his researches, which are extremely valuable.

### IV. On the Nature of the Ossification of Encysted Tumors. By *John Dalrymple*, Esq.

Mr. Dalrymple removed a small tumor from beneath the tarsal cartilage of the upper eyelid. It consisted of concentric layers of, apparently, earthy or bony matter.

"Upon examination by the microscope, the concentric layers of this tumor were found composed entirely of epithelium scales closely agglutinated together: but instead of the usual transparent and thin lamina with its central nucleus, they were thickened and hard, and contained granular earthy molecules, which could be removed by immersion in weak muriatic acid. No amorphous earthy deposit existed around or among the scales, but the whole was composed of this epithelium, opaque, of a light brown colour, with a clear and large central nucleus.

"The sebaceous or cheesy matter of encysted tumors, it is well known, consists of desquamated epithelium, somewhat disintegrated and mixed with oily globules; but here the mass may be said to have been ossified epithelium, or, in other words, these cells were filled with granular earthy deposit." 239.

Mr. Gulliver found the earthy material principally phosphate, with a trace of carbonate of lime. Mr. Dalrymple knows of no similar observation in regard to epithelium cells. Mr. Bowman, indeed, has recorded the great increase of oil globules in the cells of fatty liver; and Mr. Gulliver of biliary matter in the epithelial cells of the same organ affected with jaundice. But in both these instances the morbid condition consisted of an increase of what originally existed in a normal state.

### V. An Account of a Case in which a Foreign Body was lodged in the Right Bronchus. By Sir *Benjamin C. Brodie*, Bart. F.R.S.

This case has attracted so much attention, both with the profession and the public, that its management cannot be said to have *added* to (that would be difficult), but it certainly sustained the reputation of Sir B. Brodie, as a practical surgeon.

On the 3rd April, 1843, Mr. B. playing with a half-sovereign in his mouth, it slipped behind the tongue, and a violent fit of suffocative cough ensued. Vomiting followed. In the course of the evening he coughed moderately at intervals. Some soreness and stiffness of the throat remained for 24 hours, after which he employed himself as usual, and even entertained some friends at dinner. We find it difficult to abbreviate *usefully* the particulars which follow, and shall therefore quote them entire.

"On the 6th of April, he was again troubled with a cough. On the 7th he went on a journey into the country, and was more or less exposed to a cold north-east wind for two days and nights. The cough now became aggravated. He expectorated some mucus slightly tinged with blood, and small portions of a substance answering to the description of a thin membrane. He experienced, also, a pain in the right side of the chest, referred to a spot corresponding to the situation of the lower portion of the right bronchus.

"On the evening of the 9th of April, he took two aperient pills, one of which was rejected by vomiting some time afterwards. In the act of vomiting, he experienced a sensation as if a loose substance had shifted its place in the chest; and for some time afterwards the cough was much relieved, and the pain in the chest entirely ceased.

"On the 11th of April, the cough was again troublesome. There was little or no expectoration. At this time the chest was repeatedly examined, with the stethoscope by Dr. Seth Thompson, but no unusual sounds were detected in any part of it.

"On Monday the 17th of April, Mr. B. again went into the country, exposed to a cold easterly wind. On his return to London, the cough was again much aggravated.

"On the 18th of April, by the advice of Dr. Seth Thompson, he consulted Dr. Chambers, and afterwards myself. From the detail of the symptoms, we were all of us led to believe that the half-sovereign had passed into the trachea, and that it remained lodged in the right bronchus.

"On the 19th, this opinion seemed to be confirmed by a very simple experiment, which Mr. B. had himself made in the interval. He had placed himself in the prone position, with his sternum resting on a chair, and his head and neck inclined downwards, and, having done so, he immediately had a distinct perception of a loose body slipping forward along the trachea. A violent convulsive cough ensued. On resuming the erect posture, he again had the sensation of a loose body moving in the trachea, but in the opposite direction, that is, towards the chest.

"On the 20th, I saw the patient again, with Dr. Thompson. I now suggested that a further consultation should be held on the case; and, accordingly, on the following day there was a meeting of Dr. Chambers, Dr. Seth Thompson, Mr. Stanley, Mr. Aston Key, and myself. The chest was again carefully examined by means of the stethoscope, but no difference in the state of the respiration could be detected. The other indications of the existence of a foreign body in the air-passages, however, seemed to be so strong, that no one entertained any doubt on the subject. At this meeting it was agreed that the experiment which Mr. B. had himself made, should be repeated in a more complete manner. Accordingly, on the 25th of April, he was placed in the prone position, on a platform made to be moveable on a hinge in the centre, so that on one end of it being elevated, the other was equally depressed. The shoulders and body having been fixed by means of a broad strap, the head was lowered until the platform was brought to an angle of about 80 degrees with the horizon. At first no cough ensued; but on the back, opposite the right bronchus, having been struck with the hand, Mr. B. began to cough violently. The half-sovereign, however, did not make its appearance. This process was twice repeated, with no better result; and, on the last occasion, the cough was so distressing, and the appearance of choking was so alarming, that it became evident that it would be imprudent to proceed further with this experiment, unless some precaution were used to render it more safe.

"On the 27th of April, in a consultation of Dr. Seth Thompson, Mr. Aston Key, and myself, it was agreed that an artificial opening should be made in the trachea, between the thyroid gland and the sternum. In proposing this, we had a two-fold object; the one, that if the coin were lodged in any part from which it might be safely extracted by the forceps, this method might be had recourse to; and the other, that, if relief could not be obtained in this manner, the artificial opening might answer the purpose of a safety-valve, and enable us to repeat the experiment of inverting the body on the moveable platform, without the risk of causing suffocation. The operation was immediately performed by myself, with the assistance of Mr. Aston Key and Mr. Charles Hawkins; and on it being completed, some attempts were made, both by Mr. Key and myself, to reach the coin with the forceps introduced through the opening. The contact of the instrument with the internal surface of the trachea, however, induced on any occasion the most violent convulsive coughing. The coin was not seized, nor even felt; and our apprehensions of producing some serious mischief were such, that we did not deem it prudent, at that time, to persevere in our endeavours to remove it.

"On the 2nd of May, we again made some trials with the forceps, but always with the same result. A violent convulsive action of the diaphragm and abdominal muscles ensued, on each introduction of the instrument; and the danger of groping in the bronchus, under such circumstances, surrounded as it is by the most remarkable assemblage of vital organs in the whole body, appeared to us to be so great, that we did not think ourselves justified in proceeding further. We were the more inclined to abandon the experiment with the forceps, as we had a strong expectation that a recurrence to the first experiment, now that the safety-valve was established, would prove successful.

"On the 3rd of May, a consultation was held with Mr. Lawrence and Mr. Stanley. They entirely concurred in the views of Mr. Aston Key and myself, and it was agreed that nothing more should be attempted until Mr. B. had sufficiently recovered from the effects of what had been already done, to admit of his being again inverted on the moveable platform.

"A probe, or director, was occasionally introduced into the wound of the trachea, with a view to keep it in an open state; and, on the 13th of May, the patient having been placed on the platform, and brought into the same position as formerly, the back was struck with the hand; two or three efforts to cough followed, and presently he felt the coin quit the bronchus, striking almost immediately afterwards against the incisor teeth of the upper jaw, and then dropping out of the mouth; a small quantity of blood, drawn into the trachea from the granulations of the external wound, being ejected at the same time. No spasm took place in the muscles of the glottis, nor was there any of that inconvenience and distress which had caused no small degree of alarm on the former occasion.

"It is unnecessary to describe the progress of the case afterwards. On the 20th of May, Mr. B. had sufficiently recovered to be able to go for change of air into the country, and when I saw him, about a fortnight afterwards, the wound of the neck was nearly healed." 292.

The points of this case are so tersely and simply given in the original, that their practical effect would be marred by abbreviation, which, under such circumstances, would be mutilation. The case itself must be admitted to be highly interesting, and, as is well known, produced considerable sensation, at the time of its occurrence.

The observations of the author, appended to the narrative, are, as usual, distinguished by their practical character. Sir Benjamin Brodie remarks:—

"The different results which foreign bodies produce when admitted into the trachea, may be referred chiefly to the differences of their size, weight, and figure. If it be of large dimensions, the foreign body will be lodged, and probably impacted, in the trachea itself, causing, in the first instance, more or less obstruction to the respiration, which becomes aggravated afterwards by the too abundant secretion of mucus from the lining membrane. If it be of small size, it will descend to the lower part of one bronchus (generally the right), or even into one of the subdivisions of it, of course obstructing the respiration in a less degree. If it be of light weight, and of moderate size, having no great irregularity of figure, on every fit of coughing it will be made to ascend to the glottis, threatening, and probably at last inducing suffocation. If it be more ponderous, it will not ascend in the act of coughing, and the inconveniences which it causes, and the immediate danger, will in one respect be less." 292.

But the records of surgery amply prove that these cases are ultimately fatal, if the foreign body remains, from disease of the lungs induced by it.

The narrow space which a half-sovereign would occupy in the bronchus, explains the failure of the stethoscope as a means of detecting it. Nor is this case singular in that respect.

"I have already stated, that in making the artificial opening into the trachea, we had two objects in view; and it has been shown, that in the attainment of one of these, our success was as great as our most sanguine desires could have led us to anticipate. Although, before the opening was made, the experiment of inverting the patient on the platform was productive of a most distressing and long-continued spasm of the muscles of the glottis, no such spasm occurred afterwards. The half-sovereign escaped through the aperture of the glottis, as easily as it would have done in the dead body; and the small quantity of blood which was ejected at the same time, and which had been manifestly furnished by the granulations of the external wound, sufficiently explains how this happened: as it is not to be supposed that blood could have been drawn into the trachea without the admission of air into it at the same instant. As connected with this part of the case, it may be well here to mention, that the distressing sensations arising from congestion in the vessels of the brain, while the head was in a depending position, were immediately and completely relieved by supporting the forehead with the hand, so as to keep the occiput in some degree inclined towards the back of the neck.

"In the other object, for which the artificial opening was made, it must be confessed that we were wholly disappointed. In the dead body, with the assistance of proper forceps, there is no great difficulty in extracting a sixpence or a half-sovereign from the bronchus. But even here it is not always accomplished on the first trial. If the forceps be, as they ought to be, carefully and gently handled, the blades may actually slide over the surface of the coin without any sensation being communicated to the hand of the surgeon which will make him aware of the circumstance: or they may be passed downwards on one side of the bronchus, while the coin lies on the other. In the attempt to seize it, the forceps sometimes grasps the bifurcation of the trachea, or one of the subdivisions of the bronchus, instead of the foreign body." 295.

The depth to which the instrument must be introduced, and the convulsive action of the diaphragm and abdominal muscles, and violent cough, occasioned by its employment in the living body, sufficiently explains the difficulty. Contrary to the observations of M. Majendie on what happens in experiments on dogs, the effect, in this instance, was nearly the same, whether the instrument was directed upwards towards the glottis, or downwards towards the lungs. Sir Benjamin observes, how easy it would be to do mischief with the forceps in the bronchus, how careful the surgeon should be with them. When the foreign body is in the *trachea*, the case is different. Its removal by the forceps can then be safely and easily accomplished.

"But under all circumstances, we have a right to conclude, that an artificial opening in the trachea must contribute to the security of the patient, and that the establishment of it at an early period, is the first and most important duty of the surgeon." 297.

A sentiment which, we think, will be cordially subscribed to.

### VI. Observations on the Medicinal Properties of the Cannabis Sativa of India. By *John Clendinning*, M.D., F.R.S., Physician to the St. Marylebone Infirmary.

Looking at disease abstractedly, observes Dr. Clendinning with much justice, no indications are more important than these :—

"1. The alleviation of acute pain, whether neuralgic, spasmodic, or inflammatory, in its origin; and

"2. The securing adequate daily rest in sleep by procuring, artificially if necessary, a suspension at least of any morbid actions or conditions that might militate against refreshing repose. Almost all the suffering, and great part of the danger, of sickness may be referred to uneasy sensations of one sort or other, the irritated nervous tissues re-acting throughout the economy on the nutrient functions, deranging the elementary affinities in the blood, undermining the organic powers, and ultimately ruining the general health. Looking again at disease as we see it in clinical practice, there are no medicinal substances of more interest or importance in its treatment than such as are fitted to fulfil these two indications. In the records of medicine there are few results of professional research more striking than the beneficial effects obtained from opium in various diseases." 189.

The minor narcotics, henbane, prussic acid, &c. &c., have their uses, but none are on a par, in power, with opium. Unfortunately it has, as is well known, its drawbacks—its tendency to constipate, to arrest secretions, to affect the head. It has long, therefore, been a desideratum to find a remedy, possessed to any considerable extent of the virtues of opium, unaccompanied with its defects. Dr. Clendinning is of opinion that such a remedy is to be found in the Ext. Cannabis Indicæ.

"This agent seems, like opium, to have been known to the Orientals, and to have been in use as an article of voluptuous excitement amongst the Hindoos for a long series of ages. It was first scientifically tested, so far as I know, by Dr. O'Shaughnessy, of the Medical College, Calcutta; that gentleman was also the first, I believe, to lay the results of accurate observation before the public. The *churrus* (or resinous extract of the *gunjah* or dried Indian hemp) was found by Dr. O'Shaughnessy to possess very striking powers as an antispasmodic, as a nervine stimulant, and as an anodyne and hypnotic, and in some respects to excel opium in virtue, especially as an antispasmodic in tetanus, &c. He also observed that it was wholly, or for the most part, free from the deranging action on the stomach and bowels that so limits the utility of opium." 191.

Mr. Ley has been the first, in this country, to publish any facts upon the subject; and they lend confirmation to Dr. O'Shaughnessy's—while Dr. Clendinning has followed in the same wake.

Dr. C. reports, first, fifteen miscellaneous cases. *The first* was that of a medical man, in whom it procured sleep, without any uncomfortable consequences—*the second*, that of a woman with abdominal pains, consequent upon ovarian dropsy—*the third*, that of a nurse with sleeplessness from asthma—*the fourth*, that of a lady with a painful and inflamed cut over the wrist—*the fifth,* that of a cook with bad nights from cough and tuberculation of the lung—*the sixth,* a rheumatic case—*the seventh,* one of complicated thoracic disease—*the eighth,* one of rheumatic gout—*the ninth,* chronic cough and tuberculation of the lung—*the tenth,* disease of the lungs—*the eleventh,* cough, nervous irritation, and probably congested upon tuberculated lung—*the twelfth,* much pain in the bowels, succeeding purgative medicine, and, on another occasion, influenza and swelling of the face—*the thirteenth,* (in a medical practitioner) sleeplessness from cough—*the fourteenth,* rheumatism—*the fifteenth,* hooping-cough.

Now, in all these cases, the relief from pain, or from restlessness, or from want of sleep, or from cough, was satisfactory. In none was any unpleasant effect occasioned. Some were accustomed to the use of opium —the cannabis sativa answered as well. Some suffered from opium—they did not suffer from the cannabis. The dose was generally from ʒss. of the tincture upwards, if a single one—ten to twenty minims at intervals if repeated ones.

"*Fever Cases.*—In the course of the week commencing Feb. 24, I received into my wards half-a-dozen or more cases of spotted synochus and typhus of an asthenic character, with feeble often-jerking pulse, dry tongue, copious appearance of spots and stigmata, especially about the body; low delirium, and, in nearly all the cases, tremulousness of the tongue and limbs, amounting in several to subsultus and jactitation. They were cases in which little was indicated in the way of active medication. Quietude, ventilation, dilution, and gentle action on the abdominal functions, with cold to the head, were the means first to be thought of; and afterwards moderate stimulation was likely to become necessary. The former views were met by the effervescing draught every four hours; by shaving the head and applying cold lotions, &c.: and the latter, by wine in moderate quantity where it was deemed necessary. The fever was a low nervous one, pathologically allied to a common form of delirium tremens, and analogy suggested the use of some narcotic more efficient in conciliating sleep than any of the vinous kind. It appeared to me that opium was inadmissible. On the one hand the secretions were much deranged already, while the mischief from disturbed nights appeared by no means sufficiently pressing to warrant the use of so equivocal an agent, except in combination with mercury, aloes, or other corrective of disordered abdominal secretions. Now, the cases before me exhibited the usual tendency to diarrhœa, so that I considered it inexpedient to use any thing by which active purging might be excited. Under those circumstances I thought that I might give the hemp a trial." 205.

Three cases are given in detail. Two other (fatal ones) are mentioned. It seems unnecessary, after the general description of our author, to go into the particulars. Enough that the cannabis appeared to fulfil the indication—conciliating sleep without inconvenience. Dr. Clendinning's summing-up appears to be impartial, and is highly favourable to the medicine experimented on.

"I have no hesitation in affirming," he says, "that in my hands its exhibition has usually, and with remarkably few substantial exceptions, been followed by manifest effects as a soporific or hypnotic in conciliating sleep; as an anodyne in lulling irritation; as an antispasmodic in checking cough and cramp; and as a nervine stimulant in removing languor and anxiety, and raising the pulse and spirits; and that these effects have been observed in both acute and chronic affections, in young and old, male and female." 209.

And he adds ;—

" I have hitherto experienced no difficulty in keeping subjects of pulmonary disease under the constant operation of a narcotic, which repressed to a most important extent their mischievous cough, and secured them refreshing rest, without causing in the least degree anorexia or indigestion, or, with one or two doubtful exceptions, any inconvenient result or sensation whatever. Thus, again, I have repeatedly had a subject of articular rheumatism or severe bronchitis under the double influence at once of a diuretic-laxative medication and of an anodyne-antispasmodic; the saline solution, with or without colchicum, correcting the blood and secretions unimpeded by the narcotic, whose whole influence appeared to be expended on the tissues, seats of pain and irritation. For a third example I may refer to the use of the hemp in low fever, in securing the enjoyment of that great restorative in acute disease—viz. tranquil sleep; and producing this benefit without any neutralizing inconvenience, without causing constipation, nausea, or other effect or sign of indigestion, without headache or stupor." 210.

The per-contra would appear to be light enough :—

" The only class of cases in which I have found the hemp not to act as a competent substitute for opium, is in the intestinal fluxes, such as the diarrhœas of phthisis and of low fever in advanced stages, of old ulcerations of the bowels, &c., and in dysenteric affections. In such cases, opium is the great controlling remedy of the narcotic class, and admits of no deputy. And in such cases, happily, opium produces in judicious hands none of its inconvenient effects, and may usually be safely and freely employed." 210.

We would recommend our readers to give a trial to the medicine, and we hope it will be found to realize the expectations raised by such reports as the preceding.

### VII. On the Anatomical Characters of some Adventitious Structures, being an Attempt to point out the Relation between the Microscopic Characters and those which are discernible by the naked Eye. By *Thomas Hodgkin*, M.D.

In the 15th volume of these Transactions Dr. Hodgkin inserted a very interesting essay on compound serous cysts, their structure, mode of formation, &c., in this essay the Doctor attempted to extend the principle of the formation of these compound serous cysts to explain the structure of sarcomatous and carcinomatous tumors. Professor Müller,* who expresses his concurrence generally in the perfect correctness of Dr. Hodgkin's descriptions of, and observations on, the nature of these compound cysts, will not however at all admit the extension of the principle of the formation of these to sarcomatous and carcinomatous tumors. In the present communication Dr. Hodgkin endeavours to prove the correctness of his former views, stating that continued observation has confirmed the constant presence of the type of compound serous cysts in a class of adventitious structures which comprehends the whole family of cancerous diseases. Müller

---

* See his work on the Nature and Structural Characteristics of Cancer and other Morbid Growths.—*Reviewer*.

states, that the principle of the development of sarcomatous and carcinomatous tumors, as ascertained by observations with the compound microscope, is perfectly different in its character. Their ultimate elements, he says, are indeed cells, often permanent, so small as to be distinguishable only by employing a high magnifying power; but these cells were never observed by Dr. Hodgkin; as the very important researches regarding the function and development of nucleated cells had either not been made, or were generally unknown in this country, at the time when Dr. Hodgkin made his former communication on the subject of these cysts. We shall now present a condensed analysis of Dr. Hodgkin's paper. He first enumerates the objects which arrest attention, when a portion of one of these [adventitious] structures is placed in the field of the microscope; these are—1st. Nucleated cells of various shapes and sizes. 2. A substance having a filamentous character. 3. Granular matter without definite shape, the particles of which are often smaller than those of the nucleated cells, but by combination forming masses of comparatively large size. 4th. Very minute spherical particles resembling fat globules, and also much disposed to aggregation. 5th. Crystals, having for the most part a rhomboidal character, and often forming mackles. 6th and lastly. A transparent fluid, in which these objects are contained, and which is made evident by the motion which it permits to take place between them. He now notices each of these separately, commencing with the *nucleated cells,* both by reason of the constancy of their presence, and of the great importance now attached to them—in some specimens these cells appear to constitute the greater part of what is placed under the microscope, whilst in others they are so rare and indistinct, that it is with difficulty a cell or two can be detected amidst abundance of filamentous or amorphous granular matter—these cells are sometimes circular, sometimes oval. They often assume an elongated shape, so as to present a caudate appearance, when the prolongation takes place in one direction. Frequently prolongations take place from both ends, one or both extremities of their prolongations being occasionally bifid. These several forms in the cells are frequently met in the different varieties of disease included under the group of adventitious structures, apparently without any necessary connexion with such variety. They are most distinctly circular in specimens of cerebriform cancer, and also in those of true scirrhus. The caudate and other elongated forms are supposed to depend on the transformation of nucleated cells into a true tissue. With respect to size, also, the nucleated cells differ much, this difference being found to exist not only between cells of one specimen and another, but also between those of the same specimen. The nuclei also differ in respect of both absolute and relative size. There is also a difference in the brightness and distinctness of the nucleus. There is sometimes the appearance of a nucleolus within the nucleus, and besides one or more distinct nuclei, the cell itself may appear to be made up of minute granular matter.

He next notices the filamentous substance, which seems to have given the name to one form of these structures, described by Müller as *carcinoma fasciculatum seu desmoideum.* In some specimens little or none of this substance is traced, especially when the object is taken from the soft contents of a distinct cyst, whilst, in other examples, it appears to constitute the major part, though taken from a similar situation. Sometimes the fibrous character is distinct and well-defined, nearly resembling that of perfect cellular membrane; in other instances it is much more obscure, more nearly resembling the appearance presented by the fibrine of recently-coagulated blood, in which there is a distinct transition from granular matter. The filamentous matter may sometimes have formed a part of one of the cysts or membranes which enter into the composition of these tumors: in other instances, however, where the object is selected from a central part, filamentous matter will still be perceptible. Such cases will throw light on the question, whether fibres or filaments are of necessity produced by transition from nucleated cells. Dr. Hodgkin is induced to think, by the appearances observable in some of these growths, that fibres are not necessarily formed by the progressive elongation of perfect cells, but rather from the amorphous matter which accompanies them.*

The *granular* or *amorphous matter,* which forms one of the constituents of these tumors, is constantly present, but in very different proportions. It frequently constitutes the major part. With respect to the *spherical particles* entering into the composition of these tumors, Dr. Hodgkin is inclined to consider them as oil or fat globules. They may be found not only in the substance of a tumor, whether of scirrhous firmness, or of the soft consistence of cerebriform cancer, but also in the fluid contained in more manifest cysts. By being closely aggregated, they generally form an irregular or imperfectly globular mass. These aggregations are interesting, not merely on account of their constant presence in adventitious structures, but from their probable connexion with the fatty degeneration of normal structures. *Crystals* are very frequently, but not constantly present in these structures. They generally appear as plates made up of the aggregation of a few rhomboidal crystals. They were discovered by Müller in the discharge occasioned by the breaking-up of a cancerous tumor. On learning the discovery of these crystals by Müller, Dr. H. immediately recognized in the fact a fresh analogy between these adventitious structures and the compound serous cysts so frequently developed in the ovary or its vicinity, and in which he had seen that cholesterine may form crystalline masses resembling the purest biliary calculi of the same substance. He is therefore induced to think that these crystals may be derived from oil or fat globules by a process not of a vital character. Mr. G. Gulliver has discovered a similar crystallizable fatty matter in the coats of arteries, and in several other situations. With respect to the *transparent fluid* which forms one of the constituents of these structures, it is probably not merely derived from, but resembles the *liquor sanguinis,* and yields, like it, elementary constituents for the development of the different structures connected with it. It is generally admitted that none of the objects now enumerated, and observed to exist as constituents of adventitious structure, can be referred to for the purpose of distinguishing one form of these structures from another; nor can chemical analysis be regarded as offering

* G. Valentin states decidedly, in the form of a proposition, in his account of the development of the tissues, that "the cells and their nuclei arrange themselves in longitudinal lines; the cell-walls coalesce in lines, and, at the cost of the nuclei, form themselves into fibres."—*Reviewer.*

a more trustworthy criterion. We are, therefore, says Dr. Hodgkin, reduced to the necessity of employing an assemblage of characters to distinguish the malignant from other abnormal growths. The peculiar anatomical structure dependent on the production of compound cysts, is so constantly present, and leads to characters so essential to the correct description of individual specimens, that on these grounds he is disposed to attach much importance to it; another motive which influences him in doing so is the manifest relation which it may be shewn to bear to the nucleated cellular origin pointed out by Müller. Dr. Hodgkin assigns several causes for the difficulty of admitting the existence of a cystiform structure. The principal of these causes he states to be the production of more or less intimate adhesion between the different membranous surfaces by which a group of compound serous membranes, having completely a reflected character, may be converted into a solid mass. Maceration, which is resorted to prior to dissection, is another source of difficulty. Under the influence of this process, soft and tender structures break down and become blended with each other, and with the fluid, of which they had, at one time, formed the distinct receptacles. In this way, the well-defined vascular cysts may be converted into an irregular mass of filaments and bloodvessels—the same conversion may have occurred even during the life of the patient, by the natural progress of decay to which those structures are liable. Another source of the difficulty experienced has been taking for examination a part of the tumor so removed from the mass as not to exhibit the whole, or even the part of any principal or subordinate cyst, in which the structure in question could be demonstrated.

Dr. Hodgkin, having detected no exception to the existence of this type of cysts in the whole group of adventitious structures to which he ascribed it, naturally concluded that it depended on a law of formation common to them all, and offered as a speculation what he regarded as the probable *modus operandi* of such law. When, however, he became acquainted with the cell-theory, and with Müller's researches, which demonstrated the distinct existence of cells in the adventitious structures in question, he continued his enquiries on the subject, and it appeared to him that the adventitious structures having the type of compound serous membranes admitted of a much more satisfactory reference to the production of cells than any of the normal tissues which had been studied in their transition from cytoblasts and nucleated cells. In fact, the most perfect specimens of compound serous cysts seemed so completely to resemble a collection of nucleated cells with nucleoli, that the Dr. would have readily adopted this explanation and given up his own conjecture as to the operation of coagulation on the surface of a plastic fluid, had he not witnessed some striking facts strongly opposed to this application of the cell-theory, whilst they were in perfect accordance with that of coagulation. One of these facts is contained in his former communication to the Society, and another, and very remarkable one, is contained in the Guy's Hospital Reports, No. IV. in an account of a dissection of a patient of Dr. Ashwell's.

It being unphilosophical to admit two causes for a phenomenon, when one will suffice, Dr. Hodgkin felt somewhat staggered by the apparent necessity of admitting the two principles, viz. the cell-theory, and the coagulation-principle. Careful attention, however, to the phenomena presented by cells pointed out by Dr. Barry, in his account of the early development of the mammiferous ovum, seemed to furnish the desired solution, and not merely to combine the two modes of formation above alluded to, but to throw light on other parts of the subject. In the development of the ovum the nucleated cells appear to perform the double purpose of preparing organizable matter, hyaline, by a process of assimilation, and of giving origin to important parts by superior development, which, in this state, exert an important influence on the surrounding materials derived from other cells, inducing the coalescence of the hyaline, which they have furnished. From this it would appear that only a small number of the nucleated cells carry out their own development, but that the majority are the preparers and contributors of the pabulum of the new growth. Now the coalescence of the particles of the hyaline, appears to take place precisely in such a manner as to produce the external coagulation, on which, from observation, independently of any theory, Dr. Hodgkin has been induced to insist. Hence it is probable that some of the more enlarged cells observed in the microscopic examination of these adventitious growths, become the means of determining the coalescence around them, of the hyaline furnished by other cells, and thus account for the great tendency to the production of cysts having the compound character. With this view of the subject it is not surprising that, under the microscope, a small portion of soft matter taken from a malignant structure should often suggest a striking resemblance to the blood of some of the inferior animals, seeing that the compound of nucleated cells, granular matter and fluid, has to perform the part, in the adventitious structures, which the blood itself does in the ordinary structures. This view, Dr. Hodgkin observes, stands decidedly opposed to the theory, that the peculiar matter of cancer and other allied diseases exists as such ready-formed in the blood, from which it is eliminated at those parts at which those tumors are formed. With respect to the vascular organization of these adventitious structures, he thinks that it may be fairly concluded, from such observations as the naked eye may sometimes enable us to make, that there must be some analogy between the production of vessels in these structures, and their formation in cellular pleuritic adhesions, and other comparatively normal adventitious structures. He is of opinion that the new vessels in new parts, are the prolongation of the vessels of contiguous older parts, and not a production of independent vessels, which subsequently inosculate with those in their vicinity. Dr. Hodgkin, though he has, in accordance with the ordinary phraseology, employed the term nucleated cells in speaking of those corpuscles which present one or more brightish spots within their circumference, and have a circular, or more or less elongated figure, still has some difficulty in admitting the cellular character, as neither when they are entire, nor in their breaking-up, do they seem to exhibit any defined capsule or membrane.

The application of animal chemistry has not, up to the present time, furnished us with any sufficiently strongly-marked distinctive characters to constitute the boundaries of a class in the case of those adventitious structures, though it may indicate varieties of great practical importance. A great obstacle to our deriving a satisfactory test from chemical analysis, arises from the progressive changes which both solids and fluids undergo in the course of their production, and also when produced. Dr. Hodgkin now proceeds to answer some objections, urged by Dr. Carswell, against development through the production of compound serous cysts. Dr. Carswell observes, that there is an ambiguity as to whether the cysts produce their contents or are produced by them, and that, in the latter case, their importance is over-rated, and that the adoption of the reflected serous membrane as a type, attributes to the adventitious structure a position which, in fact, is exterior to the investing membrane, precisely as the heart is not laterally inclosed in the pericardium. Our space will not permit us to follow Dr. Hodgkin through his answers to the objections. We shall only observe that these answers are characterized by his usual tact, shrewdness, and ingenuity.

We shall now close our analysis of this valuable paper by briefly stating the conclusions which the Dr. wishes to be drawn from the observations contained in it. 1st. That continued observation has confirmed the constant presence of the type of compound serous cysts in a class of adventitious structures which comprehends the whole family of cancerous disease. 2ndly. That the microscopic examination of these tissues does not furnish perfectly conclusive tests of any particular form of adventitious structure to which a specimen may belong, but that it demonstrates the application of the nucleated cell-theory, whilst it is fatal to that of cancerous matter being formed in the blood, and eliminated at the spots at which the tumors become manifest. It therefore furnishes an important argument in favour of operations. 3rd. That to have a complete view of the mode of production of these structures, we must combine the cell-theory of Schwann and Müller, the coagulation-principle suggested by himself, and the process of organization investigated by Kiernan. 4th. That chemical analysis, however interesting, affords an imperfect criterion. 5th. That, in operating for the removal of a tumor of this class, we should leave behind none of those minute cysts which often form granules in the surrounding cellular membrane. 6th. That the infiltrated form of these diseases occurs in the structures in the neighbourhood of the purely adventitious growth, when these structures have been the seat of inflammation, and that the chances of success from operation are much diminished when such surrounding inflammation has taken place.

### VIII. Some Account of an Epidemic which prevailed at Teheran, in the Months of January and February 1842. In a Letter to Geo. Jos. Bell, M.D. Travelling Fellow of Oxford. By *C. W. Bell*, M.D. attached to H. B. M.'s Mission at the Court of Persia.

It appears that a curious new disease has appeared and spread through different parts of Teheran. A complaint in some degree similar was noticed about the same time in Baghdad.

The first case that Dr. Bell witnessed was fatal. It was that of Syad Khan. "Having stomach-ache, to which he was subject, he took ten drops of the oil of peppermint, instead of the spirit, which he was in the habit of taking, and it produced inflammation of the stomach. I applied 30 leeches to the epigastrium, by which, and other means, the inflammation was subdued. He was convalescent and in good spirits, only considerably reduced in strength, when he was suddenly attacked, about ten at night of the 20th of January, with a fit resembling epilepsy; became insensible, and died in half an hour. I saw him in this state, convulsed, the mouth drawn to one side, moaning and insensible, pulse quick, but rapidly sinking. I was wholly at a loss to account for his death, and imagined fifty things; but a *post-mortem* examination was out of the question."

Two cases, which terminated in recovery, are related next. The following is a condensed statement of the order of the symptoms in the first.

*Case.*—Mr. K., aged 40, accountant. Headache—numbness of left foot. Midnight, headache—spasms—numbness of left leg and arm. Same symptoms next day—pulse full. Recurrence of symptoms on 2nd, 3rd, and 4th nights. 5th day, epileptic fit. Two violent ones, demanding free venesection that night, followed by excruciating headache. After this, recovery. The treatment consisted of purging, quinine, bleeding.

The next case was of a very similar character. Dr. Bell, after detailing it, observes :—

"I have stated these first cases that occurred to me in detail, to show how completely I was at a loss as to the treatment which ought to be pursued. At length, three people, under my own eye, were attacked, almost at the same hour, with fits, which I could not help recognising as similar to those of which I had seen Syad Khan die. On that day, numbers had also applied to me with nervous pains, headaches, pain in the region of the heart, and nightly sleeping of the hands and feet. Venesection seemed to do harm, as well as all reduction of the system: in the two first cases, loss of blood was followed by fatal attacks. In the third and fifth, when blood had been drawn, the disease was severe. In Mr. R.'s case, quinine, as I administered it, was evidently not to be depended upon. Purgatives seemed to do no good; and in Rassool's case to do harm. What was then to be done?" 231.

He determined to try assafœtida as an antispasmodic during the fits, and to *hope* something from iron. These remedies seem to have answered completely. Several cases are related in which they appear to have acted admirably. The following is one of the shortest, but not more striking than several, indeed not so much so as some.

"M. T———, a French cavalry officer, of florid complexion, having, about the time when this complaint first made its appearance, what were thought to be threatenings of apoplexy, was bled by the advice of the medical officer to the Russian mission. After this he had a very severe fit, remaining many hours insensible; his servants continued assiduous in rubbing him, and he came to himself. He was again bled. Every night he had sensations of numbness of the leg and arm of one side, and twice again had fits, but less severe. He had suffered thus for 8 or 10 days, when he consulted me: I gave him two scruple doses of carbonate of iron; the first with 6 grains of scammony to be taken at noon, the other in the evening. That night he had no return of the numbness, nor did he experience it afterwards." 234.

Almost every one in the neighbourhood was affected for some nights with sleeping of the leg or arm of one side. Dr. Bell experienced it himself, with unnatural excitement of the heart. These all subsided after taking a dose of iron.

A curious contribution to that curious chapter—the history of epidemics.

---

